# The “Right” Definition for Post–Left Ventricular Assist Device Right Heart Failure: The More We Learn, the Less We Know

**DOI:** 10.3389/fcvm.2022.893327

**Published:** 2022-04-26

**Authors:** Shelley A. Hall, Hannah Copeland, Amit Alam, Susan M. Joseph

**Affiliations:** ^1^Baylor University Medical Center, Dallas, TX, United States; ^2^Texas A&M University College of Medicine, Dallas, TX, United States; ^3^Lutheran Hospital, Indiana University Fort Wayne, Fort Wayne, IN, United States; ^4^University of Maryland Medical Center, Baltimore, MD, United States

**Keywords:** right heart failure (RHF), left ventricular assist device (LVAD), Mechanical Circulatory Support, Academic Research Consortium, proper definition

## Abstract

Right heart failure is a major cause of morbidity and mortality following left ventricular assist device implantation. Over the past few decades, the definition proposed by the Interagency Registry of Mechanical Circulatory Support and Society of Thoracic Surgeons has continually evolved to better identify this complex pathology. We propose that the latest definition proposed by the Mechanical Circulatory Support Academic Research Consortium in 2020 will increase our recognition and understanding of this complex disease phenomenon.

## Introduction

Right heart failure (RHF) is a major cause of morbidity and mortality following left ventricular assist device (LVAD) implantation. It is estimated to occur between 9 and 42% of patients following LVAD implantation depending on the diagnostic criteria use (1). Additionally, prediction or prevention of RHF post-LVAD is challenging given the historical lack of an RHF universal definition, complicated by heterogenous derivation and validation methodologies predominantly driven by large single or multi-center studies (1).

After continuous-flow LVADs were first approved for destination and bridge to transplantation strategies, the fourth annual Interagency Registry of Mechanical Circulatory Support (INTERMACS) in 2012 concluded that RHF “represents a major challenge to the successful application of continuous-flow technology and constitutes a major thrust of future INTERMACS research” (2). This declaration continues to hold true in 2022 despite improved VAD technology with 2-year survival nearing that of heart transplantation (3, 4). In fact, the most recent INTERMACS annual reports omitted the RHF post-LVAD in their outcomes, noting varying definitions and lack of consistency making an analysis unreliable (4–7). Herein, we propose that the latest definition proposed by the Mechanical Circulatory Support Academic Research Consortium (MCS-ARC) to diagnose RHF-LVAD will help mitigate the ongoing challenges encountered by the heart failure community in this decade-long quest to find the right definition for RHF post-LVAD.

## Classifications and Evolution of the Definition

The INTERMACS started as a partnership among the National Heart, Lung, and Blood Institute, hospitals, and industry in 2006 (8). The first adverse event definition for RHF by INTERMACS is shown (9) (Table 1). This definition did not accurately describe the specific objective criteria needed to identify the signs or symptoms suggestive of RHF nor incorporate the biomarker or laboratory assessment in their diagnosis criteria. In addition, a cutoff of central venous pressure (CVP) of 18 mmHg may be too non-specific and not capture the degrees of RHF post-LVAD. In fact, many studies published their outcomes of “RHF post-LVAD” not using this specific definition but rather a modified definition of the initial proposal by INTERMACS giving recognition to the varying degrees of disease presentations. Arigiriou et al. reported, using inotropes for more than 14 days or discharge from hospital to home, on inotropes with specific inotropic drug dose criteria to further define a more precise definition for RHF post-LVAD (9, 10). Kormos et al. also reported outcomes based on the timing of inotropic initiation and duration following LVAD implantation, acknowledging the role of varying mechanisms that may be causing early and late occurrences of RHF post-LVAD (11). Importantly, none of these studies utilized the sole definition proposed by INTERMACS. More precise and clinically applicable definitions were clearly needed.

**Table 1:** 2008 INTERMACS definition for right heart failure.

**Symptoms and signs of persistent right ventricular dysfunction**
Central venous pressure > 18 mmHg with a cardiac index <2.0 L/min/m^2^
Right Ventricular Assist Device Implantation OR use of inhaled nitric oxide or inotropic therapy for a duration of more than 1 week at any time after LVAD implantation
Absence of elevated left atrial or pulmonary capillary wedge pressure (>18 mmHg), tamponade, ventricular arrythmias or pneumothorax

In 2014, a refined definition to include the time frame from surgery and more specific diagnostic criteria were proposed by INTERMACS (Table 2). This definition incorporated documentation of a lower CVP of 16 mmHg by heart catheterization or elevated CVP by imaging or physical exam assessments. Furthermore, manifestations of elevated CVP were needed either through physical exam (e.g., edema), imaging (e.g., ascites), or through specific laboratory markers (e.g., elevated bilirubin or creatinine). This definition was more inclusive; however, the heart failure community continued to use varying definitions to define RVF post-LVAD. Parameters such as low mixed venous oxygen saturation levels, varying degrees of elevated CVP, CVP/wedge ratio, need for right-sided VAD, assessments of tricuspid regurgitation, tricuspid annular motion on echocardiography among other clinical variables were included to define RVF (1, 12). Despite an updated and more inclusive definition in 2014, the application for a sole definition of RVF remained heterogenous by the LVAD community—appropriately—given recognition of patients with clinical findings suggestive of RVF that may not be included in the 2014 definition.

**Table 2:** 2014 INTERMACS definition for right heart failure.

**Definition: Symptoms or findings of persistent right heart failure characterized by BOTH of the following:**
1. Documentation of elevated central venous pressure by: - Direct measurement with right atrial pressure > 16 mmHg OR - Findings of significantly dilated inferior vena cava with absence of inspiratory variation by echocardiography OR - Clinical findings of elevated jugular distention at least halfway up the neck in an upright patient
2. Manifestations of elevated central venous pressure characterized by: - Clinical findings of peripheral edema (>2+ either new or unresolved) OR - Presence of ascites or palpable hepatomegaly on physical examination or diagnostic imaging OR - Laboratory evidence of worsening hepatic congestion (total bilirubin > 2.0 mg/dl) or renal dysfunction (creatinine > 2.0 mg/dl)
3. If the patient meets definition of both criteria above then a severity scale for right heart failure will be graded utilizing post implant inotropes, inhaled nitric oxide or intravenous vasodilators, need for right ventricular assist device and timing from surgery.

In 2018, the INTERMACS was acquired by the Society of Thoracic Surgeons. In 2020, the MCS-ARC proposed a more expanded and inclusive definition of RHF post-LVAD (13), recognizing the varying degrees of phenotypical presentations. This definition is focused based on timing from LVAD implantation and acuity of up-escalation of mechanical or non-mechanical support (Table 3). An elevated CVP is not needed for the diagnosis, rather the pieces of evidence of RVF through a physical exam, imaging, broadened elevated biomarkers or hemodynamic parameters from right heart catheterization were now included. The significant limitations that existed in the prior definitions are now improved to become more sensitive for disease recognition.

**Table 3:** 2020 Academic Research Consortium definition for right heart failure.

**Early acute right heart failure**	**Early post-implant** **right heart failure**	**Late right heart failure**
Need for implantation of right ventricular assist device at time of left ventricular assist device implantation	(A) Need for implantation of right ventricular assist device <30 days of left ventricular assist device implantation OR (^*^B) Failure to wean from inotropic support or inhaled nitric oxide within 14 days following LVAD implantation or having to initiate this support within 30 days of implant for a duration of at least 14 days OR (C) Death occurring in patients within 14 days of LVAD implant who have not received an RVAD but who remain on inotropes or vasopressors at the time of death and meet criteria for the diagnosis of Right Heart Failure on the basis of the above clinical findings	(A) Need for implantation of right ventricular assist device >30 days of left ventricular assist device implantation OR (^*^B) Hospitalization that occurs >30 days post-implant and which requires intravenous diuretics or inotropic support for at least 72 h.
	^*^ For Criteria B: At least two of the following must be present: - Ascites - Peripheral edema (>2+) - Elevated central venous pressure (>16 mmHg) - Elevated jugular venous pressure atleast half way up the neck in an upright patient OR At least one of the following must be present: - Renal failure with creatinine > 2 × baseline value - Liver injury with at least 2 × upper limit normal in AST/ALT - Total bilirubin > 2.0 - SVO2 <50% - Cardiac index <2.2 liter/min/m^2^ - Elevated lactate > 3.0 mmol/liter - Reduction in pump flow of >30% from previous baseline in absence of cardiac tamponade, tension pneumothorax or other mechanical causes.	

## Discussion

Right heart failure (RHF) post–durable left ventricular assist device (LVAD) remains its Achilles heel, given its myriad of phenotypical presentations both in terms of timing post-implant and severity. The definition of RVF as an outcome has been traditionally heterogeneous in both time frames (acute, early, and late) and diagnostic criteria, with varied elements such as the INTERMACS definition, RVAD implantation, and prolonged inotrope/vasodilator dependence. Unfortunately, even contemporary landmark clinical trials (e.g., Momentum 3) that report RHF following LVAD have modified the INTERMACS 2014 definition to include RVAD implantation, need for inhaled nitric oxide, or inotropic therapy for >1 week to make it less subjective (3). More recently, Rame et al. used the 2014 INTERMACS definition to report an incidence of late RHF at 5% for mild and moderate RHFs and 0.2% for severe RHF in 2021 (14). The actual incidence of late RHF post-LVAD is likely much higher than that reported in this study given the more sensitive and inclusive definition of RVF reported by the MCS-ARC. To our knowledge, no study has reported outcomes of RVF post-LVAD utilizing the 2020 contemporary definition recommended by the MCS-ARC.

Albert Einstein famously said that *if he had 1 h to save the world, he would spend 55 min defining the problem and only 5 min finding the solution*. As an LVAD community, we need a contemporary, objective definition of RHF that is not dependent on documentation of subjective physical exam findings and limited laboratory results. The 2020 MCS-ARC definition of RHF, includes laboratory (lactate, SVO2, liver, and renal function), clinical (need for RVAD, inotropes or inhaled nitric oxide within 14 days, low pump flow), and hemodynamic parameters (low cardiac index) in addition to the physical exam to define the severity of RHF, is certainly a step in that direction. Or is it?

Is the 2020 MCS-ARC definition the final evolution or will this too become obsolete? More importantly, will it be utilized by the LVAD community or will we continue to modify definitions according to our traditional behaviors? Should we evolve the definition to further prognosticate the severity of RHF and include other hemodynamic variables such as pulmonary artery pulsatility index, impact of severe valvular heart disease, or response and/or resistance to diuretics? Only one thing is sure in our field: the more we learn, the less we know.

## Data Availability Statement

The original contributions presented in the study are included in the article/supplementary material, further inquiries can be directed to the corresponding author.

## Author Contributions

All authors listed have made a substantial, direct, and intellectual contribution to the work and approved it for publication.

## Conflict of Interest

SH was consultant/advisor for Abbott, Abiomed, Medtronic, CareDx, Natera. HC was speaker for Abbott; spouse is a consultant for Syncardia. SJ was speaking Honoraria for Abbott. The remaining author declares that the research was conducted in the absence of any commercial or financial relationships that could be construed as a potential conflict of interest.

## Publisher's Note

All claims expressed in this article are solely those of the authors and do not necessarily represent those of their affiliated organizations, or those of the publisher, the editors and the reviewers. Any product that may be evaluated in this article, or claim that may be made by its manufacturer, is not guaranteed or endorsed by the publisher.
